# IgG exacerbates genital chlamydial pathology in females by enhancing pathogenic CD8 ^+^ T cell responses

**DOI:** 10.1111/sji.13331

**Published:** 2023-10-13

**Authors:** Charles W. Armitage, Connor P. O'Meara, Emily R. Bryan, Avinash Kollipara, Logan K. Trim, Danica Hickey, Alison J. Carey, Wilhelmina M. Huston, Gavin Donnelly, Anusch Yazdani, Richard S. Blumberg, Kenneth W. Beagley

**Affiliations:** ^1^ Centre for Immunology and Infection Control and School of Biomedical Sciences Queensland University of Technology (QUT) Brisbane Queensland Australia; ^2^ Drop Bio Ltd, School of Biotechnology and Biomolecular Sciences (BABS) University of New South Wales Sydney New South Wales Australia; ^3^ School of Life Sciences University of Technology (UTS) Sydney Ultimo New South Wales Australia; ^4^ Queensland Fertility Group (QFG) Brisbane Queensland Australia; ^5^ Division of Gastroenterology, Department of Medicine Brigham & Women's Hospital, Harvard Medical School Boston Massachusetts USA

**Keywords:** asymptomatic, *Chlamydia*, FcRn, IgG, transmission

## Abstract

*Chlamydia trachomatis* infections are an important sexually transmitted infection that can lead to inflammation, scarring and hydrosalpinx/infertility. However, infections are commonly clinically asymptomatic and do not receive treatment. The underlying cause of asymptomatic immunopathology remains unknown. Here, we demonstrate that IgG produced during male infection enhanced the incidence of immunopathology and infertility in females. Human endocervical cells expressing the neonatal Fc Receptor (FcRn) increased translocation of human IgG‐opsonized *C. trachomatis*. Using total IgG purified from infected male mice, we opsonized *C. muridarum* and then infected female mice, mimicking sexual transmission. Following infection, IgG‐opsonized *Chlamydia* was found to transcytose the epithelial barrier in the uterus, where it was phagocytosed by antigen‐presenting cells (APCs) and trafficked to the draining lymph nodes. APCs then expanded both CD4^+^ and CD8^+^ T cell populations and caused significantly more infertility in female mice infected with non‐opsonized *Chlamydia*. Enhanced phagocytosis of IgG‐opsonized *Chlamydia* significantly increased pro‐inflammatory signalling and T cell proliferation. As IgG is transcytosed by FcRn, we utilized FcRn^−/−^ mice and observed that shedding kinetics of *Chlamydia* were only affected in FcRn^−/−^ mice infected with IgG‐opsonized *Chlamydia*. Depletion of CD8^+^ T cells in FcRn^−/−^ mice lead to a significant reduction in the incidence of infertility. Taken together, these data demonstrate that IgG seroconversion during male infection can amplify female immunopathology, dependent on FcRn transcytosis, APC differentiation and enhanced CD8 T cell responses.

## Graphical abstract

IgG‐opsonized *Chlamydia* had enhanced epithelial transcytosis, where it is uptaken by antigen‐presenting cells which subsequently stimulated T cells causing significant infertility in mice. To underpin this finding, FcRn (responsible for IgG transcytosis) knockout mice had altered chlamydial shedding and T cell‐mediated pathology.

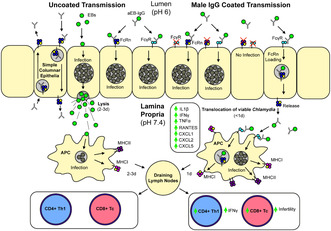

## 1 INTRODUCTION

Urogenital *Chlamydia trachomatis* infections of the male and female urogenital tracts are the world's leading sexually transmitted bacterial infection with an estimated 127 million new cases each year.^1^ Despite widespread public awareness and education, infections continue to rise, quadrupling in the USA in the last two decades,^2^ and costing an estimated US$ 2 billion per annum.^3^ Chlamydial infection is asymptomatic in males (30%‐60%) and females (70%‐90%) allowing continued dissemination without treatment,^4^ likely owing to recurrent infections and the influence of the high allelic variability of human major histocompatibility complexes (MHC).^5^ If detected, *Chlamydia* spp. are sensitive to azithromycin and doxycycline antibiotics, but if left untreated acute asymptomatic infection can lead to chronic inflammation and severe sequelae including fallopian tube scarring and occlusion, pelvic inflammatory disease (PID), ectopic pregnancy and infertility.^6^ A widely used model of *C. trachomatis* infection is vaginal infection of female mice with the mouse‐specific strain *C. muridarum*, which leads to an ascending infection, causing scarring and occlusion of the oviduct (Fallopian tube) and hydrosalpinx (infertility).^7^ Because of the severe sequelae associated with *C. trachomatis* infections, it is widely accepted by the scientific community that there is an urgent requirement for a chlamydial vaccine protecting against pathology.

Antibodies are glycoproteins that are a crucial arm of the adaptive immune response that are produced in response to infection or immunization and are responsible for the immunity obtained from most successful vaccines. Antibodies have multiple functions including not only steric blocking of pathogen attachment to host cells and subsequent invasion, but also enhancing phagocytosis of pathogens by neutrophils, macrophages and dendritic cells. Immunoglobulin G (IgG) is the most abundant immunoglobulin in the blood accounting for 80% of all antibodies, and its concentration in mucosal secretions is dependent on the tissue type. In the female reproductive tract, IgG is found within secretions at a range of 10‐467 and 1‐200 μg mL^−1^ in vaginal and cervical/uterine secretions respectively.^8^ In male ejaculate, there is between 16 and 33 μg mL^−1^ of IgG (depending on the volume)^8^ that is secreted by B cells into the circulation and is transcytosed into the mucosal lumen by the epididymis, prostate and accessory organs of the male reproductive tract.^8^ Until relatively recently delivery of IgG into the lumen was thought to be mediated by passive flux, it is now suggested that IgG is transported across epithelial cell barriers into the mucosa of mammals by FcRn.^9^, ^10^

The FcRn is expressed throughout the mammalian body on a diverse range of cell lineages, for example, epithelia, endothelia, hepatocytes, podocytes, syncytiotrophoblasts, neutrophils and antigen‐presenting cells.^9^ FcRn facilitates passive immunity in embryos/neonates via delivery of maternal IgG to the offspring across the placenta and from breastmilk in the neonatal GIT. FcRn is also expressed throughout the body in the gastrointestinal, respiratory and urogenital tracts throughout life.^9^, ^11^

Unlike other Fc gamma receptors (FcγR_I‐IV_) which bind IgG and IgG‐bound immune complexes (IgG:IC) near the Fc hinge region of IgG, FcRn reversibly binds IgG:IC and free IgG at the CH2‐CH3 domain under acidic conditions (pH 5‐6.5) and releases IgG at neutral pH (pH 7.4), facilitating bidirectional transport of IgG across epithelial cells into and out of the lumen allowing rapid delivery of antigen (or pathogens) to immune cells in the lamina propria.^9^, ^12^, ^13^, ^14^ As both the male and female reproductive tracts express FcRn^11^, ^15^ and have acidic lumens in the epididymis,^16^ prostate,^17^ vagina and cervix,^18^ the potential for FcRn to deliver IgG‐bound pathogens to immune cells in the lamina propria is of scientific interest.

*Chlamydia* spp. are obligate intracellular bacteria with a biphasic lifecycle that not only infect mucosal epithelia of the urogenital tracts, but also infect and replicate within leukocytes including neutrophils, macrophages, dendritic cells, modulating how the immune system responds to infection^19^, ^20^ but also trafficking infection to unconnected tissues.^21^ Unique from other intracellular bacteria, *Chlamydia* spp. replicate within a parasitophorous vesicle termed the inclusion which secretes proteins that interfere with normal host processes including blocking apoptosis,^22^ inhibiting binding of host endocytic pathway and lysosomal recruitment,^22^ as well as MHC‐I and MHC‐II presentation.^23^, ^24^ Unlike many other intracellular pathogens, the entry of *Chlamydia* into cells can be active or passive.^25^ Like *Chlamydia* spp, the intracellular pathogens *Toxoplasma gondii*, *Mycobacterium bovis* and *Legionella pneumoniae* can infect cells and avoid host cell lysosomal degradation.^26^ However, IgG opsonization of these pathogens prior to internalization enhances phagocytosis via binding of IgG to FcγR, which internalize IgG:ICs and targets them for lysosomal degradation.^26^ Conversely, phagocytosis of IgG‐opsonized chlamydial elementary bodies (EBs) enhances uptake but continues to avoid fusion with host cell lysosomes allowing inclusion formation and replication.^12^, ^27^, ^28^ Similarly, it has been demonstrated that IgG specific for the ubiquitously expressed chlamydial antigen Major Outer Membrane Protein (MOMP) can bind *Chlamydia* EBs and enhance uptake in FcγR and FcRn‐dependent mechanisms.^12^, ^27^, ^28^, ^29^ As antibodies are generated in response to chlamydial infection, we sought to determine if IgG from males infected with *Chlamydia* could enhance transcytosis and immunopathology in naïve females following sexual transmission.

## 2 MATERIALS AND METHODS

### 2.1 Ethics statement

This study was approved by the Queensland University of Technology Animal Ethics Committee (QUT UAEC # 1400000606). All animals were killed by intraperitoneal injection with sodium pentobarbital (200 mg kg^−1^). Human seminal plasma samples were collected from Queensland Fertility Group (QFG) (Brisbane, QLD, Australia) under accordance with the QFG ethics (02.14) and QUT Human Research Ethics Committee (UHREC #1500000765). Female human sera were collected with approvals from Prince Charles Human Research Ethics Committee (EC2809), Ipswich and West Moreton Health Services District (10‐09), Gold Coast Hospital District (200893), Cairns Sexual Health Clinic (HREC/09/QCH/4‐554), QUT (1 300000003 and 080000268) and IVF clinics based in Brisbane (UC Health HREC 1314) and Melbourne (HREC number 12099).

### 2.2 Animals

Mice (BALB/c) were sourced from the Animal Resource Centre (ARC; Canningvale, WA, Australia) at 6 weeks of age and given food and water ad libitum. Female BALB/c mFcRn^−/−^ mice were backcrossed >10 generations from C57BL/6 mFcRn^−/−^ (B6.Fcgrt^tm1Dcr^/DcrJ, #003982) mice sourced from The Jackson Laboratory (Bar Harbour, ME, USA).

### 2.3 Cells and Chlamydia

Endocervial epithelia (SiHa) (Cat # HTB‐35) were purchased from the American Type Cell Culture (ATCC; Manassas, VA, USA). Cells were cultured in RPMI 1640 supplemented with 10% heat‐inactivated fetal calf serum, 100 μg mL^−1^ streptomycin sulfate, 50 μg mL^−1^ gentamycin and 1× Glutamax (ThermoFisher) (RPMI+). *C. muridarum* (Weiss strain) and plasmid‐cured *C. muridarum* (Nigg strain; CM3.1)^39^ were a generous gift from Dr Catherine O'Connell (UNC Chapel Hill) and were cultured from McCoy cells and purified as previously described.^53^ *C. trachomatis* serovar D (VR‐885) and serovar E (VR‐348B) were purchased from the ATCC. *Chlamydia* spp. were UV‐inactivated for 2 × 20 min, and biotinylated using NHS‐biotin (Sigma Aldrich) overnight at 4°C in 0.1 mol L^−1^ NaHCO_3_ pH 9 (0.125 mg NHS‐Biotin:1 mg protein). Mouse bone marrow was collected as previously described,^54^ differentiated into macrophages over 6 days using RPMI+ supplemented with 0.2 μm‐filtered 10% L929 cell supernatant or into dendritic cells using media containing 20 ng mL^−1^ recombinant mouse GM‐CSF (Lonza, Mt Waverly, VIC, Australia) and 20 ng mL^−1^ recombinant mouse IL‐4 (Lonza) as previously described.^54^

### 2.4 Antibodies

IgG was purified using Protein G (Genscript, Piscataway, NJ, USA) from the pooled (n = 10) cardiac bleed sera of male BALB/c mice that had previously been infected with 10^6^ inclusion forming units (IFUs) of *C. muridarum* (Weiss) for 10 weeks as previously described.^34^ Naïve mouse IgG (Sigma Aldrich, Castle Hill, NSW, Australia) was used as control IgG. IgG was also purified from human blood using Protein G. Human seminal plasma samples were collected from QFG as part of routine sperm collection for IVF. Purified mouse IgG and human IgG and seminal plasmas were stored at −80°C. Mouse anti‐IncA IgG was generated by immunizing mice as previously described.^12^ Rabbit anti‐mouse FcRn‐cytoplasmic tail (mFcRn‐CT) and rabbit anti‐human FcRn‐cytoplasmic tail (hFcRn‐CT) was produced as previously described.^31^

### 2.5 Transwells

**FIGURE 1:**
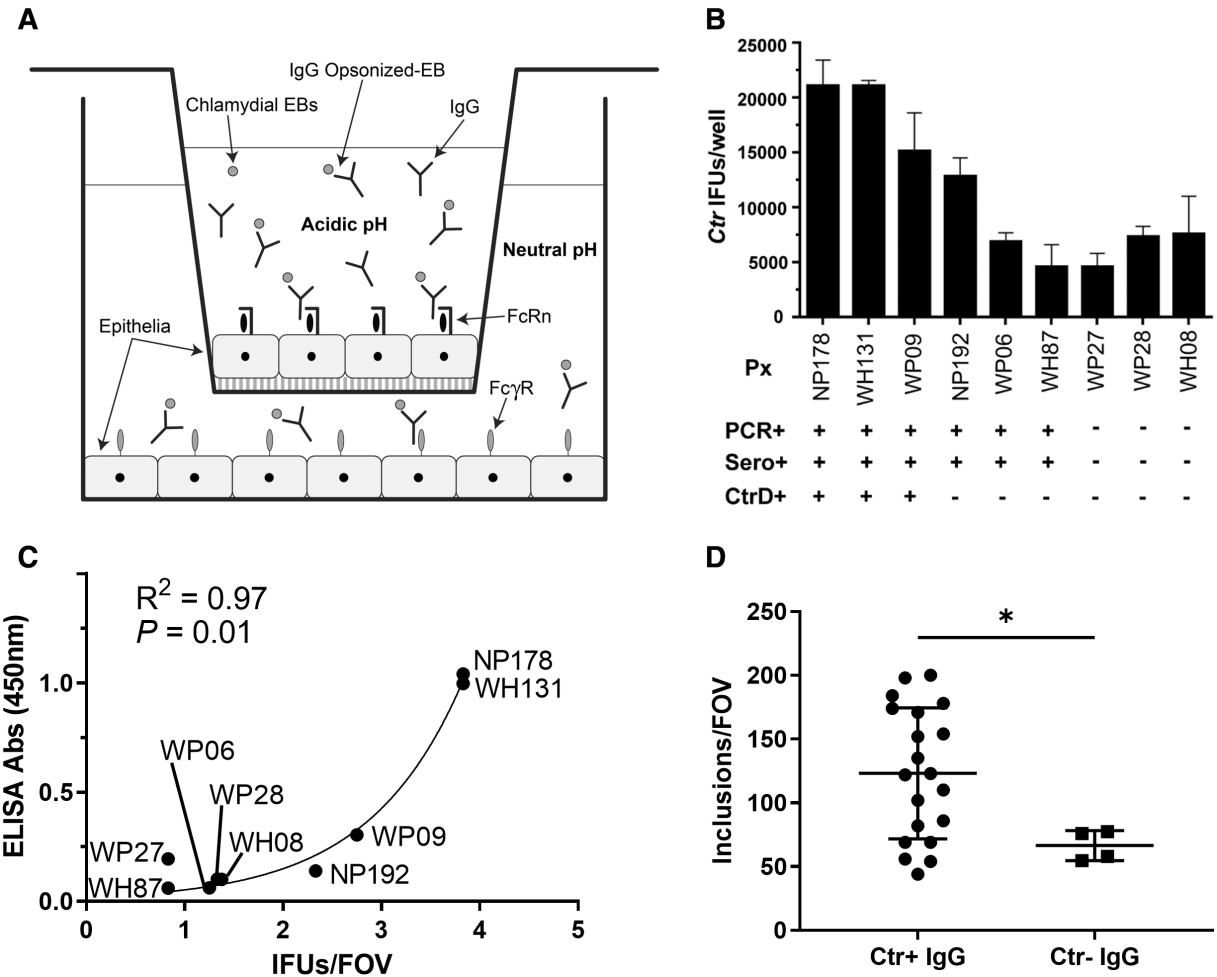
IgG‐opsonization enhances chlamydial translocation in human female reproductive epithelial cells. Human female reproductive epithelial cells (SiHa cells apically and basally) were seeded and allowed to polarize over 5‐6 days. Purified immunoglobulin G (IgG) from *Chlamydia trachomatis* D (CtrD+) seroconverted (Sero+) participants (confirmed by polymerase chain reaction [PCR+]) or naïve human serum was used to opsonize CtrD elementary bodies, which were applied to the apical well under physiological pH of 6.5, then inclusions (IFU) in the basal well were quantified after 48 h by immunocytochemistry (A, average IFU per field of view [FOV] is displayed in D). The same purified IgG was used in a direct enzyme‐linked immunosorbent assay (ELISA) against CtrD EBs to correlate opsonization with participant (Px) infection status (B). Lastly, CtrD‐IgG‐positive and negative seminal plasma was used to opsonize CtrD EBs, which were used to infect SiHa cells at pH 6.5, then IFU were quantified after 48 h by immunocytochemistry (C). N = 3/infection status group, data representative mean and SD of triplicate experiments (A). ELISA data in (B) was assessed for correlation using non‐linear regression (exponential growth) and the Spearman correlation test. N = 20/CtrD+ IgG group, N = 4/CtrD‐IgG group (C). Graphs and statistical analysis (unpaired *t* test) was generated in GraphPad Prism (v9), *P < .05 (D).

Transwells used were 24‐well format (Falcon Cat # 353504) with 3 μm inserts (Falcon Cat # 353181) seeded with 5 × 10^4^ SiHa cells per apical insert and basolateral well (Figure 1A) as previously described.^12^ Cells were incubated for 6 days with media changes every 2 days in both the apical and basal chambers. Cells were cultured in RPMI 1640 supplemented with 10% heat‐inactivated fetal calf serum, 100 μg mL^−1^ streptomycin sulfate, 50 μg mL^−1^ gentamycin and 1× Glutamax (ThermoFisher) (RPMI+). Cells in the apical chamber were then infected at MOI 1 with *C. trachomatis* serovar D, which were pre‐incubated with antibody. Opsonization for Figure 1A was achieved by incubating EBs with purified IgG (as above, 100 μg mL^−1^ in HBSS [containing CaCl_2_ and MgCl_2_, Gibco]) on ice for 1 h, then EBs were added to apical well at pH 6 by buffering the culture media with MES (50 mM). Opsonization for Figure 1C was achieved by incubating EBs with whole seminal plasma (as above) on ice for 30 min, then EBs were added to the apical well.

### 2.6 Intravaginal infections

Female mice (6‐8 weeks) were hormonally synchronized by subcutaneous injection of 2.5 mg depot medroxyprogesterone (Depo Provera, Pfizer, New York, NY, USA) 1 week prior to challenge as previously described.^34^ *C. muridarum* (500 IFUs per 20 μL) was incubated with 5 or 50 μg mL^−1^ of infected male IgG (EB IgG) or naïve IgG for 1 h on ice prior to infection. Mice were intravaginally infected with 500 IFUs Cmu + IgG under anaesthesia with ketamine (Parnell Laboratory, Alexandria, Australia; 100 mg kg^−1^)/xylazine (Bayer, Leverkusen, Germany; 10 mg kg^−1^). In vivo depletion of CD8β^+^ T cells (Clone: 53‐5‐8, IgG1, 250 μg intraperitoneal, 50 μg intravaginally; BioXCell, West Lebanon, NH, USA) or IgG1 isotype control (anti‐HRP; BioXCell) was performed the day prior to challenge. Depletion efficiency was assessed previously and the same method was used in this study.^34^

### 2.7 Bone marrow RNA transcription

Murine bone marrow macrophages or dendritic cells were infected with IgG‐opsonized *C. muridarum* (αEB‐IgG 100 μg mL^−1^), or *C. muridarum* + naïve IgG (100 μg mL^−1^) and incubated at 37°C 5% CO_2_ for 2, 4, 6 or 24 h. RNA was extracted using Trizol (ThermoFisher) and 10 μg of glycogen carrier protein (Cat # AM9510, Applied Biosystems, Foster City, CA, USA) according to the manufacturer's instructions. Complementary DNA was synthesized using High Capacity Reverse Transcriptase Kit (Applied Biosystems) (Cat. # 4368814) as per the manufacturer's instructions. Each reaction contained 10 ng of cDNA, 1 μM of forward/reverse primers (Sigma Aldrich), 200 μM dNTPs, 1.5 mM MgCl_2_, 1× buffer, 0.15× SYBR green and 5 U of Platinum Taq polymerase (ThermoFisher) made up to a final volume of 20 μL using sterile nuclease‐free water. Reverse transcription PCR was performed using the Corbet Rotorgene Q (Qiagen). Primers for mouse CXCL1, CXCL2, CXCL5, TNF, IL1β and IFNγ mRNA were used as previously described. Mouse Dendritic and Antigen Presenting Cell RT^2^ Profiler PCR Array (PAMM‐406Z) (Qiagen) was performed as per the manufacturer's instructions on an ABI 7900 HT plate PCR (Applied Biosystems).

### 2.8 *Chlamydia*‐specific proliferation and cytokine production

**FIGURE 2:**
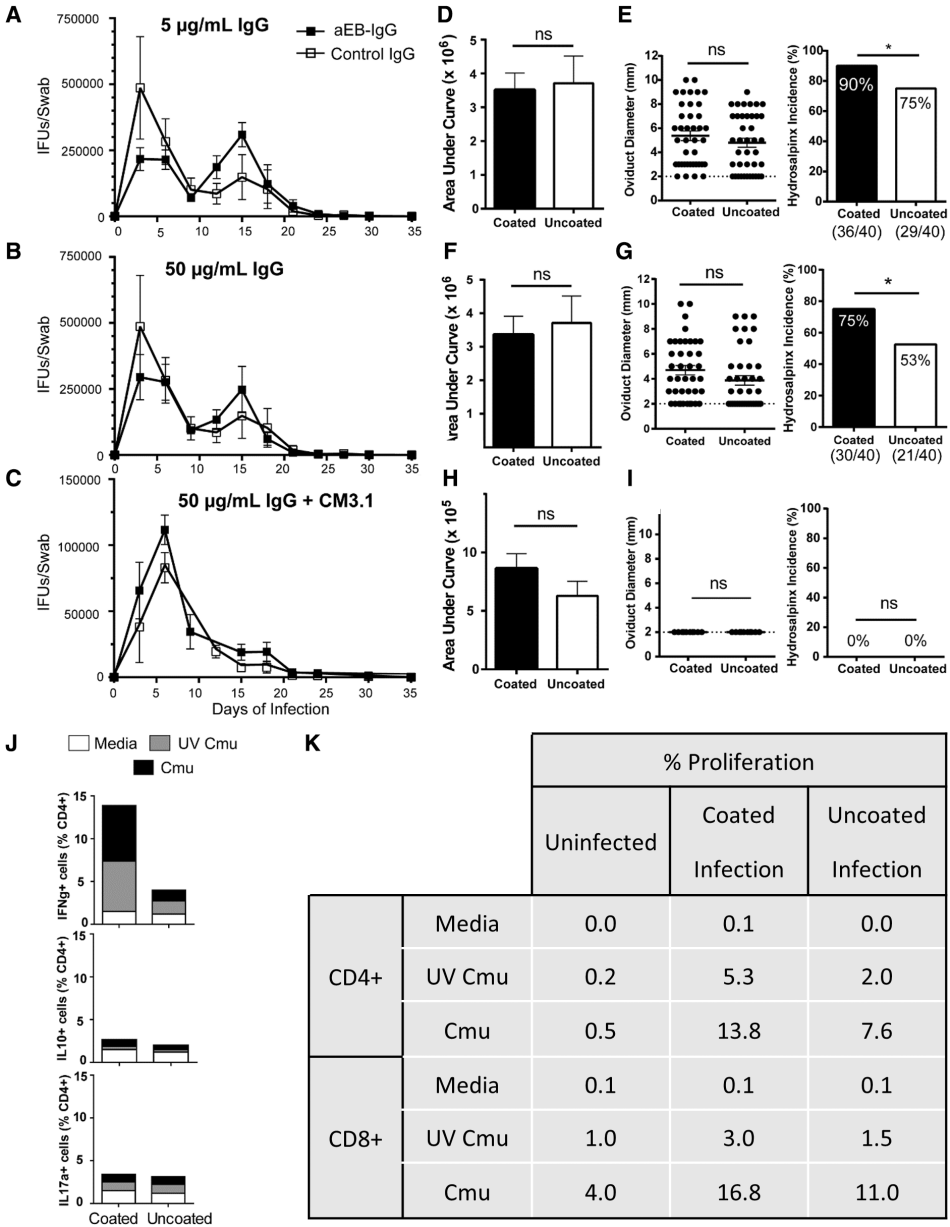
Vaginal inoculation with optimized *Chlamydia* enhances hydrosalpinx and T cell activation. Female BALB/c mice were infected with 5 (A–C) or 50 (D–E) μg mL^−1^ of anti‐*C. muridarum* (Cmu) elementary body immunoglobulin G (αEB‐IgG)‐opsonized or non‐opsonized *C. muridarum* (Weiss), or plasmid‐cured (CM3.1) *C. muridarum* (F–I) for 35 days and vaginal shedding (and total burden displayed as area under curve) and hydrosalpinx measured (n = 10 mice [20 oviducts]/group). (J) Pooled caudal and lumbar lymph nodes (five mice/group) were taken and stimulated in vitro with ultraviolet (UV)‐inactivated Cmu or live Cmu (MOI = 1) for 12 h and CD4^+^ T cell intracellular cytokine production was determined by flow cytometry. (K) Splenocytes from infected mice were labelled with CSFE and stimulated in vitro with UV Cmu or live Cmu for 3 days. CD4^+^ and CD8^+^ T cell proliferation was determined by flow cytometry. N = 10 mice/group, 20 datapoints shown for oviduct measurements as two oviducts per mouse, from one experiment. Graphs and statistical analysis generated in GraphPad Prism (v7), data representative of mean and SD of one experiment. Hydrosalpinx severity (oviduct diameter) was compared between groups with unpaired *t* tests and hydrosalpinx incidence (presence‐absence) data were analysed with chi‐square, chlamydial shedding data analysed with unpaired *t* tests, *P < .05.

**FIGURE 3:**
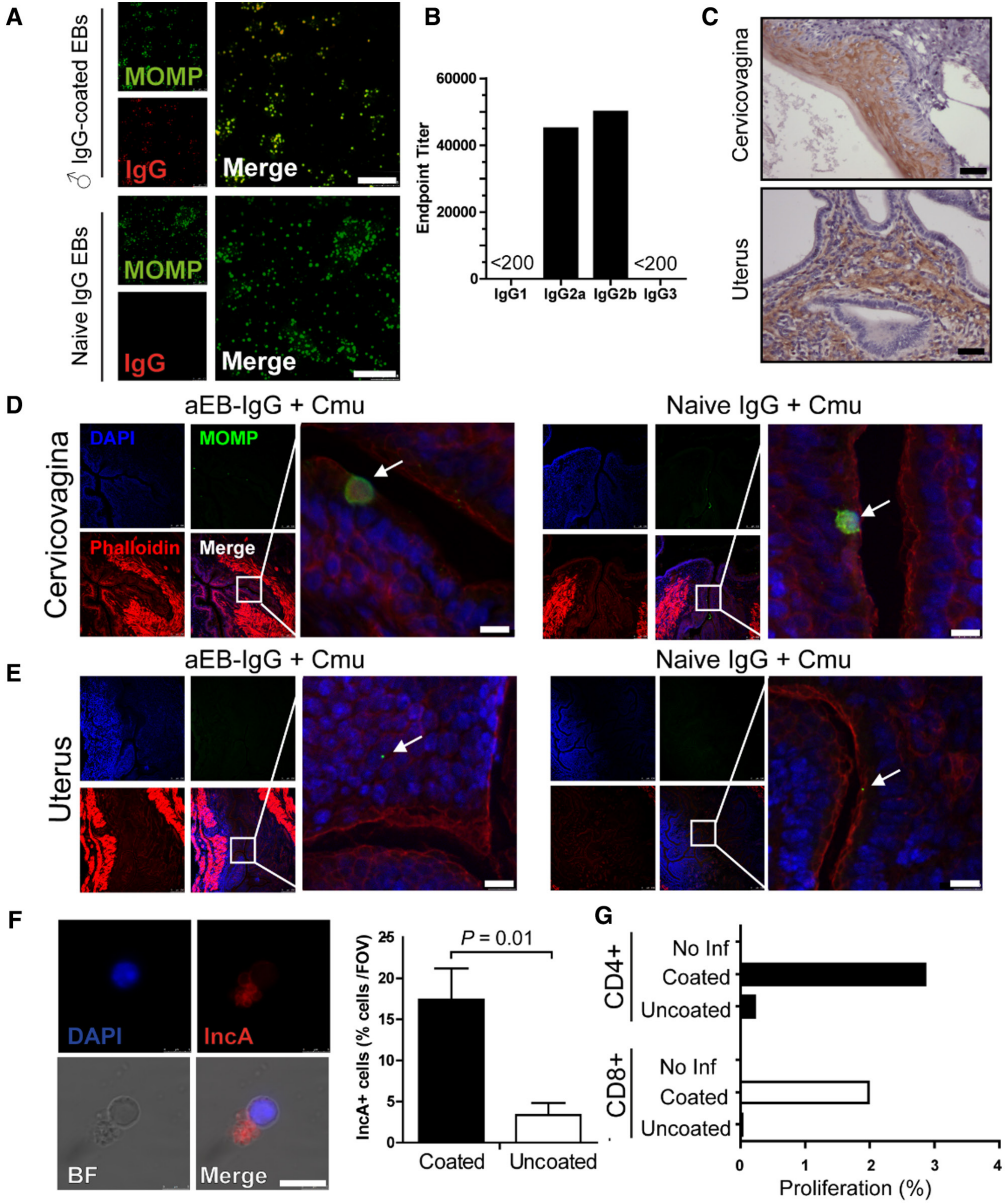
Opsonization of *Chlamydia* facilitates translocation and lymph node migration. A, UV‐inactivated *C. muridarum* (Cmu) was immobilized on ELISA plates and male anti‐*C. muridarum* elementary body immunoglobulin G (αEB‐IgG) or naïve IgG added. EBs and IgG were detected with sheep anti‐major outer membrane protein (MOMP) IgG and visualized with anti‐sheep IgG‐AF488 and anti‐mouse IgG‐AF555. B, Isotypes of αEB‐IgG were determined by direct ELISA with ultraviolet (UV)‐inactivated *C. muridarum* and anti‐mouse IgG1‐3‐HRP. C, IHC of uninfected mouse cervicovagina and uterine horn tissues was performed to detect FcRn infection. D, Cervicovaginal and E uterine horn tissues of mice infected with *C. muridarum* for 24 h were frozen, cryosectioned and IHC was performed with anti‐MOMP, counterstained with phalloidin for cytoskeleton and DAPI for nucleus. F, 24 h post‐infection, lymph nodes were collected, homogenized, fixed and stained with DAPI and anti‐inclusion membrane protein A (IncA) IgG and detected with anti‐mouse IgG‐AF555. G, CD4^+^ and CD8^+^ T cell proliferation was determined by flow cytometry without in vitro stimulation. White arrows identify inclusion formation. Images are representative of 10 fields of view per mouse. Scale bars = white (10 μm), black (20 μm). Fluorescent images captured with SP5 confocal microscope (Leica Biosystems); DAB images captured with using light microscopy. Flow cytometry performed using Aria III (BD Bioscience). Graphs and statistical analysis (unpaired *t* test) generated with GraphPad Prism (v7), data mean and SD. N = 3 (A–F) or five (G, pooled nodes) mice/group, from one experiment.

Lymphocytes were stained with carboxyfluorescein succinimidyl ester (CFSE) (Sigma Aldrich; 5 μM) for 5 min at room temperature. Splenocytes were cultured in RPMI 10% FCS, 100 μg mL^−1^ streptomycin sulfate, 50 μg mL^−1^ gentamycin, 1× Glutamax and 50 μM b‐mercaptoethanol. Labelled splenocytes were seeded into U‐bottom 96 well plates (5 × 10^4^ cells/well) and stimulated with media containing live (MOI 1) or UV‐inactivated (10 μg mL^−1^) *C. muridarum* for 72 h at 37°C with 5% CO_2_ (Figure 2) or cultured in media without stimulation (Figure 3). For cytokine production, non‐CSFE‐labelled cells incubated for 12 h in medium containing brefeldin A (Sigma Aldrich; 10 μg mL^−1^) prior to intracellular cytokine staining. Cells were washed with 2% (v/v) FCS/PBS before blocking with αCD16/CD32 IgG (Clone: 24G2) for 15 min at 4°C. Cells were stained with αCD3 IgG‐APC Cy7 (Clone 145‐2C11, Cat # 423112, Biolegend, San Diego, CA, USA), αCD4 IgG‐V450 (Clone: RM4.5, Biolegend) and αCD8α IgG‐PE Cy7 (Clone: 53.6‐7, Cat # 25‐0081, eBioscience, San Diego, CA, USA) in a volume of 50 μL for 15 min at 4°C. Cells were washed with 2% FCS/PBS before fixing with 4% (w/v) paraformaldehyde in PBS for 10 min at 4°C. Fixed cells were then permeabilized with Perm/Wash (Cat # 554723, BD Bioscience, Franklin Lakes, NJ, USA) for 15 min at 4°C. Permeabilized cells were stained with αIFN‐γ IgG‐ APC (Clone: SMG1.2, Cat No. 554413, BD Bioscience), αIL‐10 IgG‐FITC (Clone: FES5/16E3) and αIL‐17a IgG‐PE (Clone: TC11‐18H10, Cat # 559502, BD Bioscience) diluted in Perm/Wash buffer for 40 min at 4°C. Stained cells were analysed on a FACSAria III (BD Bioscience) using flowjo version 10.2 software (Tree Star Inc, Ashland, OR, USA). Gating strategy is shown in Figure S1.

### 2.9 Immunohistochemistry

Paraffin‐embedded tissues were sectioned and stained as previously described but with some modifications.^34^ Primary antibody rabbit anti‐mouse FcRn‐CT (5 μg mL^−1^), and secondary goat anti‐rabbit IgG‐HRP (1:1000) (Southern Biotechnology). For cryosectioned samples, tissues were snap frozen in Tissue‐Tek Optimum Cutting Temperature (OCT) Compound (ProSciTech, Kirwan Australia), and sectioned (5 μm) using a CM3050 S Research Cryostat (Leica Biosystems). Slides were fixed in ice‐cold acetone for 10 min at 4°C, air‐dried for 10 min at room temperature and blocked with 5% (v/v) FCS/PBS for 1 h at room temperature. Slides were stained with sheep anti‐*C. muridarum* MOMP sera (made in‐house)^55^ for 1 h at room temperature. Slides were washed three times with PBS for 5 min, and stained with donkey anti‐sheep IgG‐AlexaFluor 488 (Thermo Fisher), Phallodan‐AF594 (Thermo Fisher) and DAPI (Thermo Fisher) for 1 h at room temperature. Slides were washed three times with PBS, mounted with Prolong Gold (Thermo Fisher) and analysed with a Leica TCS SP5 confocal microscope (Leica Biosystems).

### 2.10 Gross oviduct pathology

The oviduct diameter was measured on day 35 post‐infection to assess the incidence and severity of gross pathology. The severity was determined by increase in size from healthy oviducts (2 mm), and incidence of hydrosalpinx was the total number of occluded oviducts (unilateral and bilateral).

### 2.11 Power calculations and statistical analysis

Sample sizes were determined a priori using a one‐tailed, proportions: inequality, two independent groups Fischer's exact test in g*power 3.1.7 software (Institute of Experimental Psychology, Dusseldorf, Germany). Significance is represented using the symbol *, with *P > .05, **P > .01 and ***P > .001. Significance between vaginal shedding was determined using Student *t* tests. The significance of the severity and incidence of hydrosalpinx was determined using Student t test and chi‐squared tests, respectively, performed using graphpad prism version 7 (GraphPad, La Jolla, CA, USA). The total IFU/swab (the chlamydial infectious burden) was quantified, and the difference between groups analysed, as area under curve, using graphpad prism version 7. As area under curve assesses the bacterial burden, only infected groups are displayed, as the non‐infected control has a zero datapoint.

## 3 RESULTS

### 3.1 IgG opsonization of *Chlamydia* enhances transcytosis across epithelia

Previous studies have demonstrated a role for IgG‐mediated enhanced phagocytosis and transcytosis of opsonized antigen and pathogens across mammalian mucosal epithelia^12^, ^13^, ^30^, ^31^, ^32^, ^33^; hence, we sought to determine if this phenomenon was applicable in the context of chlamydial infections. Purified human IgG was used to opsonize *C. trachomatis* EBs, which was applied to polarized pH 6.5 endometrial/cervical epithelial cells (SiHa) using a previously established Transwell cell culture model (Figure 1A). *C. trachomatis* serovar D‐specific IgG derived from actively infected (PCR‐positive) and chlamydial‐positive seroconverted donors was found to enhance translocation of EBs across the polarized epithelia to the basolaterally seeded cells (SiHa), whereas donors who were PCR negative and had no *C. trachomatis* D‐specific IgG had minimal translocation of *C. trachomatis* D (Figure 1B). As there was a trend towards current infection (PCR) and *C. trachomatis‐*specific IgG seroconversion (validated by ICC), direct ELISAs of purified IgG against *C. trachomatis* D EBs were performed, and statistical analysis of translocation revealed a significant correlation between *C. trachomatis* serovar D EB‐specific IgG titre and translocation across epithelia into the basolateral chamber (Figure 1C). Because chlamydial infection of males also induces *Chlamydia*‐specific IgG in the male reproductive tract, which could potentially enhance infection in females, seminal plasma‐opsonized EBs were applied to the apical surface (SiHa) and allowed to infect. Seminal plasma from *C. trachomatis*‐IgG+ donors significantly (*P* < .05) enhanced infection of basolateral cells (SiHa), compared to *C. trachomatis*‐IgG donors (Figure 1D). As FcRn is important for IgG transcytosis and translocation of IgG‐opsonized antigen in mucosal tissues^12^, ^13^, ^30^, ^31^, ^32^, ^33^ and has a pivotal role in IgG transcytosis in the mammalian female reproductive tract,^9^ we sought to further investigate the role in infection and pathology in a FcRn^−/−^ mouse model.

### 3.2 Opsonization of *Chlamydia* with male immune IgG enhances female pathology

Male infection with *C. muridarum* leads to a chronic non‐resolving infection in the male reproductive tract, with a chronic viable infection detectable in the prostatic fluid, prostate, penis and testes for at least 10 weeks,^34^ and up to 6 months in the testes.^35^ Male infection leads to production of systemic anti‐chlamydial EB IgG antibodies detectable in the sera within 4 weeks.^36^ Male to female sexual transmission of *Chlamydia* in mice is not possible, as female infections require hormone synchronization with progesterone making the females unresponsive to breeding.^34^, ^37^, ^38^ Thus, to simulate sexual transmission of *Chlamydia* from chronically infected males (>10 weeks) which have large concentrations of αEB‐IgG, or acutely infected (<2 weeks) (naïve IgG) prior to seroconversion, chlamydial EBs were incubated with IgG, and then female mice were intravaginally infected with 500 IFUs as this has previously been shown to be a biologically relevant dose.^34^ To control for the range of total IgG in male ejaculate, EBs were incubated at the low (5 μg mL^−1^) and high concentrations (50 μg mL^−1^) prior to infection. Infection of female mice with IgG‐opsonized EBs did not significantly alter the kinetics of the infection, nor did it significantly change total chlamydial burden at either the low or high IgG concentrations (Figure 2A,B), indicating poor in vivo neutralization by αEB‐IgG. When female mice were infected with less pathogenic and virulent plasmid‐cured EBs, αEB‐IgG also failed to alter the chlamydial infection kinetics or pathology (Figure 2C). After 6 weeks of infection, oviduct (fallopian tube) occlusion severity and frequency of occurrence (incidence) was recorded (Figure 2D–I). Female mice that had been infected with IgG‐opsonized EBs had no significant change in the severity of blockage, but interestingly had significantly more incidence of hydrosalpinx, indicating infertility, at both the low and high IgG concentrations (Figure 2E,G). Infection of females with opsonized less virulent, plasmid‐cured EBs did not spontaneously induce the development of hydrosalpinx (Figure 2I) as expected.^39^ Caudal and lumbar LN cells collected from mice infected with IgG‐opsonized Cmu contained significantly more IFNg‐secreting CD4^+^ cells, when stimulated in vitro with live or UV‐inactivated Cmu, compared to cells collected from non‐opsonized Cmu‐infected mice (Figure 2J). IL‐17a and IL‐10‐secreting CD4^+^ cell numbers were not significantly different between the groups (Figure 2J). Splenocytes from animals infected with IgG‐opsonized Cmu contained greater numbers of proliferating CD4 and CD8 cells, following in vitro stimulation with Cmu (Figure 2K). Taken together, these results indicate that opsonization of *Chlamydia* with immune IgG prior to infection does not affect the kinetics or resolution of infection, but significantly enhances the incidence of the immunopathological sequelae and T cell responses to infection.

### 3.3 Translocation of IgG immune complexes and immune activation

Chlamydial infection of male mice induces a chronic upper reproductive tract infection lasting 6 months,^35^ which facilitates a robust and consistent adaptive immune response. Opsonization of EBs with male IgG did not significantly alter the kinetics of infection (Figure 2A–D) but enhanced the incidence of hydrosalpinx (Figure 2E,F), we therefore sought to determine the mechanism of action. Purified male αEB‐IgG was found to co‐localize with immobilized chlamydial EBs indicating purification did not affect the ability of IgG to bind infectious *Chlamydia* (Figure 3A). Conversely, naïve mouse IgG did not bind chlamydial EBs, indicating antigen‐specific binding, and not Fc/Fab‐mediated binding observed in other bacteria. Anti‐chlamydial EB (αEB)‐IgG (specifically IgG_2a_ and IgG_2b_) were detected in the sera of male mice infected for 10 weeks, indicative of a strong IgG_2a_:IgG_1_ Th1 bias during male infection consistent with previous studies (Figure 3B).^12^, ^36^ In female mice hormonally synchronized with DepoProvera, FcRn expression was detectable in the vagina and uterine horns prior to infection (brown DAB staining, Figure 3C) consistent with previous studies.^11^

We have previously shown that opsonization of *Chlamydia* with IgG from MOMP‐immunized animals can also enhance pathology by translocation across epithelia, dependent on FcRn expression.^12^ To determine where this was happening in vivo, female mice were infected for 24 h allowing EB uptake and inclusion development within one cycle of infection. IHC of females infected for 24 h showed chlamydial infection of stratified squamous epithelia of the cervicovagina in both the EB‐IgG‐opsonized and control IgG *Chlamydia* groups indicating luminal infection of cells at the mucosal barrier (Figure 3D). Interestingly, in the mouse, uterus replicating *Chlamydia* were detected below the simple columnar epithelial layer in the laminar propria of IgG‐opsonized EBs infected mice, but not in non‐opsonized‐infected mice suggesting translocation of IgG immune complexes occurred in the uterus (Figure 3E).

In addition to stromal fibroblasts, leukocytes (particularly antigen‐presenting cells [APCs]) are present in the lamina propria which phagocytose antigens, migrate along afferent lymph vessels to draining lymph nodes and activate the adaptive immune response. To determine if replicating *Chlamydia* had been trafficked to the draining lymph node, caudal and lumbar lymph nodes were collected 1 day post‐infection. Lymphatic cells were stained using an anti‐inclusion membrane antibody which detects replicating *Chlamydia*. In mice infected with IgG‐opsonized EBs, there were significantly more IncA^+^ (actively replicating) cells in the draining lymph nodes (Figure 3F) indicating viable and replicating *Chlamydia* had been trafficked to the lymphatics inside APCs. Three days after infection, lymph node cells were collected, labelled with CFSE and cultured in vitro for three additional days. CSFE‐proliferation of CD4^+^ and CD8^+^ T cells (Figure 3G) was determined by flow cytometry. CD4^+^ and CD8^+^ T cells isolated from the draining lymph nodes of mice infected with IgG‐opsonized *Chlamydia* proliferated more than 10‐fold more than cells from mice infected with non‐opsonized infections, suggesting APCs in the lymph node were rapidly presenting antigen and stimulating rapid T cell responses. Taken together, these data suggest that EBs opsonized with IgG from infected males can rapidly translocate infectious EBs across simple epithelia, infecting and replicating inside APCs which rapidly traffic to draining lymph nodes and enhance CD4^+^ and CD8^+^ T cell proliferation.

### 3.4 Opsonization of *Chlamydia* alters APC activation

**FIGURE 4:**
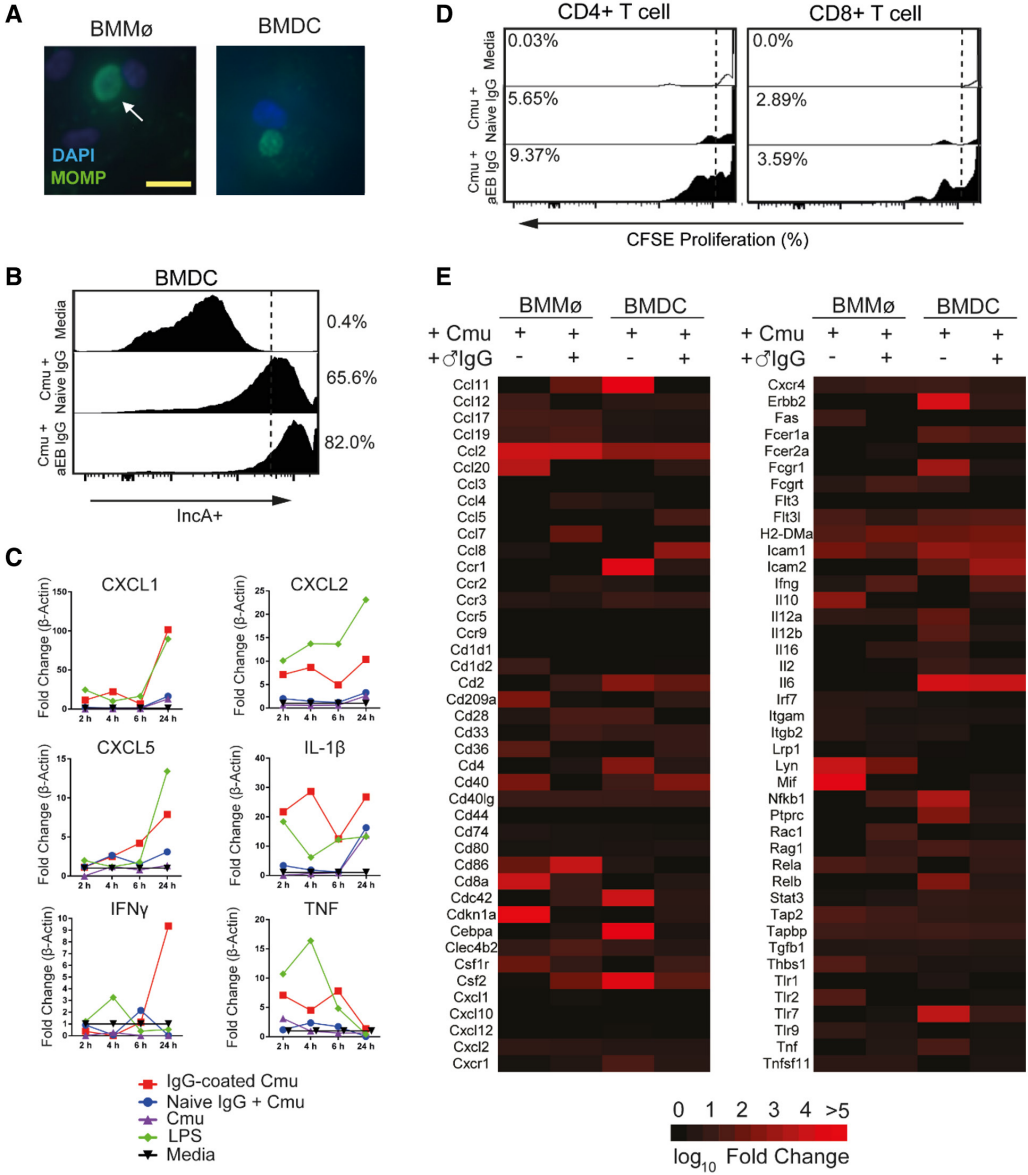
Opsonization of *Chlamydia* alters APC activation and antigen presentation. A, Bone marrow‐derived macrophages (BMM_Ø_) and dendritic cells (BMDC) were infected with *C. muridarum* (Cmu, MOI1) for 24 h, stained for major outer membrane protein (MOMP) expression and imaged with a fluorescent microscope. B, BMDCs were infected for 24 h with opsonized (100 μg mL^−1^) or non‐opsonized *C. muridarum*, fixed and stained with anti‐inclusion membrane protein A (IncA) mAb‐PE and analysed by flow cytometry (Aria III, BD Bioscience). C, BMDCs were infected (+/− IgG opsonization) for 24 h, and then incubated with CSFE‐labelled splenocytes from *C. muridarum*‐infected female mice (day 35 post‐infection) for 3 days analysed for CD3^+^ CD4^+^ and CD8^+^ expression by flow cytometry. D, BMDCs were seeded into 24‐well plates and were infected with *C. muridarum* (MOI = 1) (+/− opsonization) and mRNA collected over 24 h. qRT‐PCR was performed on a Rotorgene Thermocycler. E, Bone marrow‐derived macrophages (BMM_Ø_) and bone marrow‐derived dendritic cells (BMDCs) were seeded into a 6‐well plate and infected with *C. muridarum* (MOI = 1) (+/− opsonization) for 24 h. RT‐PCR was performed using a Dendritic and Antigen Presentation RT2 Array (Qiagen) analysed on 7900HT FAST ABI. White arrows indicate chlamydial inclusion. Scale bar = 10 μm. Graphs generated with GraphPad Prism (v7). N = 5 mice/group, pooled, from one experiment.

*Chlamydia* spp. can infect APCs altering their phenotype.^11^, ^20^ To investigate the role opsonizing chlamydial EBs in males αEB‐IgG has on female APCs, bone marrow macrophages (BMM_Ø_) and dendritic cells (BMDC) were generated and infected with IgG‐opsonized or non‐opsonized *C. muridarum* (Figure 4A). Phagocytosis of *Chlamydia* did not result in uptake and killing of chlamydial EBs, and flow cytometry for inclusion membrane production demonstrated replication in both opsonized and non‐opsonized EBs, with enhanced production of IncA (active replication marker) in BMDCs infected with opsonized EBs (Figure 4B). Infection of BMDCs also led to increased in vitro stimulation of CD4^+^ and CD8^+^ T cells from splenocytes collected from previously infected female mice (Figure 4C).

To determine the kinetics of mRNA translation during infection, BMDCs were infected with non‐opsonized or opsonized *C. muridarum* and samples taken over a 24 h period (Figure 4D). Infection of BMDCs with EBs opsonized with male IgG enhanced expression of chemokines CXCL1, CXCL2, CXCL5 and pro‐inflammatory cytokines TNF, IL1β and IFNγ compared to uninfected or non‐opsonized controls.

To determine the extent and change in APC differentiation, BMM_Ø_ and BMDCs were infected with opsonized or non‐opsonized EBs for 24 h and mRNA analysed with an Antigen Presentation RT Array (Figure 4E). Infection of BMM_Ø_ with IgG‐opsonized EBs upregulated mRNA transcription of chemokines and cytokines *Ccl2/MCP‐1* (highest, 7245‐fold), *Ccl12/MCP‐5*, *Ccl20/MIP‐3A* and *Il10*; antigen presentation *Cd1d2*, *Cd8α* (highest, 6015‐fold), *Cd36*, *Cd40*, *Cd40lg/Tnfsf5*, *Cd86*, *Cd209a/DC‐SIGN* and *Icam*; and signal transduction *Cdkn1a*, *Csf1r*, *Fas*, *Flt3*, *Lyn* (highest, 5515‐fold), *Mif* and *Rela*. Conversely, infection of BMM_Ø_ with IgG‐opsonized *Chlamydia* upregulated mRNA transcription of chemokines and cytokines *Ccl11*, *Ccl2/MCP‐1* (highest, 5537‐fold), *Ccl7/MCP‐3* and *Ifnγ*; antigen presentation and chemotaxis *Cd8α*, *Cd28*, *Cd33*, *Cd40lg/Tnfsf5*, *Cd86* (highest, 4842‐fold), *Cdc42*, *Clec4b2*, *Csf2/GM‐CSF*, *Cxcr4*, *H2‐DMa/MHC‐2*, *Icam1* and *Rac1*; and signal transduction *Lyn* (highest, 118‐fold), *Nfkβ1* and *Rela*.

Dendritic cells are considered pivotal in antigen presentation and induction of adaptive immunity and behaved considerably different to BMM_Ø_ following uptake of IgG‐opsonized or non‐opsonized *Chlamydia*. Infection of BMDCs with non‐opsonized Cmu, assessed via gene array, upregulated mRNA for chemokines and cytokines *Ccl2/MCP‐1*, *Ccl11/Eotaxin* (highest, 47 673‐fold), *Ccr1*, *Il6*, *Il12α*, *Il12β*, *Tnf* and *Csf2/GM‐CSF*; antigen processing and chemotaxis *Cd2*, *Cd4*, *Cd28*, *Cd40*, *Cdc42* (highest, 6880‐fold), *Cxcr1*, *Cxcr4*, *Icam1* and *Fcgr1/CD64*; and signal transduction *Cebpa* (highest, 28, 565‐fold), *Erbb2*, *Nfkβ1*, *Ptprc*, *Rag1* and *Relb*. Conversely, infection with IgG‐opsonized *Chlamydia* led to the upregulation of mRNA for chemokines and cytokines *Ccl2*, *Ccl5/RANTES*, *Ccl8/MCP‐2*, *Il6* (highest, 5928‐fold), *Csf2/GM‐CSF* and *Ifnγ*; antigen processing and chemotaxis *Cd2*, *Cd40*, *H2‐DMa/MHC‐2*, *Icam1* and *Icam2* (highest, 744‐fold); and signal transduction *Flt3I* (37‐fold). Taken together, opsonization of *Chlamydia* with male αEB‐IgG prior to infection significantly alters the antigen processing and activation of both macrophages and dendritic cells, resulting in enhanced inflammatory and migratory signalling when APCs phagocytose male IgG‐opsonized *Chlamydia*.

### 3.5 FcRn trafficking of immune complexes mediates enhancement of pathology dependent on CD8 ^+^ T cells

**FIGURE 5:**
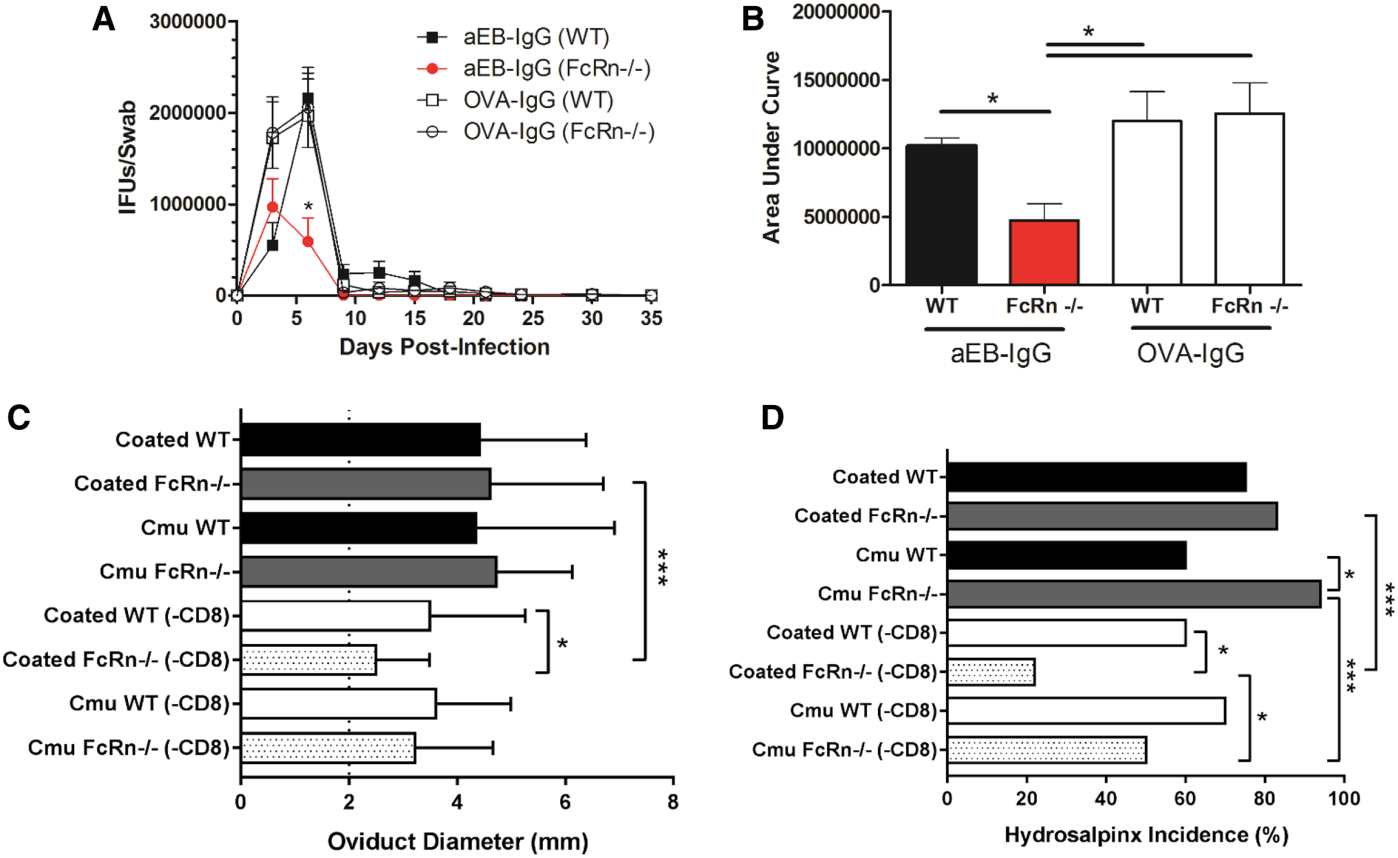
FcRn‐mediated translocation enhances CD8 immunopathology. (A, B), Female wild‐type (WT) and FcRn knockout (−/−) mice were infected with *Chlamydia muridarum* (Cmu, +/− opsonization with 50 μg mL^−1^ IgG) for 35 days and vaginal shedding determined by culture (N = 5‐10, in one experiment), with total burden displayed as area under curve. (C, D), Female WT or FcRn^−/−^ mice were depleted or mock depleted of CD8β^+^ T cells (Clone: 53‐5‐8, IgG1, 250 μg intraperitoneal injection, 50 μg intravaginally; BioXCell, West Lebanon, NH, USA) 1 day prior to being infected with opsonized (male anti‐*C. muridarum* IgG [αEB‐IgG] 50 μg mL^−1^) or non‐opsonized (naïve IgG 50 μg mL^−1^) *C. muridarum*. Hydrosalpinx occurrence and severity (diameter, mm) was measured 35 days post‐infection. (N = 9‐10 mice/group from one experiment). Graphs (mean and SD) and statistical analysis (two‐way‐ANOVA) was completed in GraphPad Prism (v7), *P < .05, ***P < .001.

Opsonization of *Chlamydia* prior to infection was found to enhance pathology in vivo (Figure 2); thus, we sought to address what was driving the enhancement of pathology using knockout mice and depletion studies. It has previously been demonstrated that cross presentation of IgG:IC by dendritic cells can enhance CD8^+^ T production,^31^ and CD8^+^ T cells generated in response to vaginal chlamydial infection are also immunopathogenic as evidenced by the lack of hydrosalpinx in OT‐I mice.^40^ To determine if FcRn was mediating enhancement of immunopathology, we infected WT and FcRn^−/−^ mice with IgG‐opsonized or non‐opsonized *Chlamydia*. Unlike previous infections of WT mice (Figure 2), opsonization of *Chlamydia* with IgG prior to infection led to significant reductions in the kinetics, peak, duration and total burden of *Chlamydia* in FcRn^−/−^ mice alone (Figure 5A,B). However, WT and FcRn^−/−^ did not have any significant differences when infected with non‐opsonized *Chlamydia* demonstrating the reduction in burden in FcRn^−/−^ mice was dependent on opsonization. To determine the role of FcRn and CD8^+^ T cells, the mice were depleted (or mock depleted) of CD8^+^ T cells 24 h prior to infection and hydrosalpinx measured after 6 weeks. In WT mice that had been depleted of CD8^+^ T cells or mock depleted and then had been infected with opsonized or non‐opsonized *Chlamydia*, there was no significant reduction in severity or incidence of hydrosalpinx suggesting a limited role for CD8^+^ T cells during the early stages of infection (Figure 5C,D). Interestingly, in FcRn^−/−^ mice infected with opsonized *Chlamydia*, there was a significant reduction in incidence of pathology if CD8^+^ T cells were also depleted prior to infection (94%‐22%). This protection was also significantly more than in FcRn^−/−^ mice infected with non‐opsonized *Chlamydia* and depleted of CD8^+^ T cells (50%‐22%) suggesting EB‐IgG opsonization prior to infection improved the protection afforded from depleting cytotoxic T cells. In WT and FcRn^−/−^ mice depleted of CD8^+^ T cells and infected with opsonized *Chlamydia*, there was a significant reduction in pathology in the absence of FcRn suggesting in the absence of IgG:IC transcytosis, CD8 depletion early during female infection is more protective against hydrosalpinx.

## 4 DISCUSSION

Urogenital chlamydial infections affect both males and females, and the deleterious immunopathology adversely afflicting females is studied widely; yet, chlamydial infections are asymptomatic in 70%‐90% of females^41^ leading to under‐diagnosis, continued dissemination and chronic inflammation that can cause scarring and infertility. Similarly, males are asymptomatic in 30%‐50% of *C. trachomatis* urogenital infections^41^ and while the immunopathology can lead to chronic infections of the prostate and testes, male infections and the implications on infertility are poorly understood and understudied. As chronically infected male mice can harbour an active infection >10 weeks^34^ and is culturable for at least 6 months in the testes,^35^ and seroconvert with a Th1‐bias within 1 month of infection, we sought to determine if IgG antibodies generated in response to a chlamydial infection in males could influence the outcome of infection and immunopathology in females, mimicking sexual transmission. While secretory IgA may also play a role in steric blocking of *Chlamydia* and the host response to infection,^42^, ^43^ FcRn does not bind IgA and there is generally low titre IgA within seminal fluid (relevant for mimicry of sexual transmission) compared to IgG,^44^ leading this study to focus on IgG.

Monoclonal and polyclonal IgG antibodies generated following immunization with MOMP have been demonstrated to enhance infectivity of *C. trachomatis* and *C. muridarum* in vitro via FcγR‐mediated uptake on epithelial cells,^12^, ^28^ including uptake by FcRn.^12^ We have also previously demonstrated that MOMP‐specific IgG can translocate infectious *Chlamydia* across epithelial barriers infecting underlying cells, dependent on FcRn‐mediated translocation under acidic luminal conditions.^12^ This phenomenon has also been reported in HIV infections with FcRn‐mediated translocation of IgG‐opsonized HIV across polarized endometrial epithelial cells monolayers, also dependent of acidic luminal conditions.^13^ The 19 serovars of *C. trachomatis* were originally differentiated based on serotyping of IgG binding to MOMP, and the divergence of the MOMP serovars is derived predominantly from variability of surface‐exposed domains of MOMP on the surface of EBs. Thus, we sought to determine if the phenomenon of enhanced chlamydial infection observed with MOMP‐specific IgG in vitro and in vivo was repeatable using IgG antibodies generated during a natural male infection, and then infecting female mice with opsonized *Chlamydia*.

To determine if translocation of infection could also occur in vivo, naïve female mice were infected with *C. muridarum* opsonized with male IgG, and tissues were taken 1 day later. Consistent with human in vitro studies, opsonization of *C. muridarum* failed to prevent infection of female mice, with chlamydial inclusions detected in both the cervicovaginal and endometrial epithelium. Interestingly, and consistent with the in vitro findings, IgG‐opsonization of *Chlamydia* facilitated translocation across the uterine epithelia into the lamina propria, but not in the vaginal tissues. Significantly more replicating (IncA^+^ staining) *Chlamydia* was detectable in the lymph nodes draining the female reproductive tract, in females infected with opsonized EBs. Furthermore, lymph node homogenates collected from female mice infected with opsonized EBs were also able to rapidly facilitate CD4^+^ and CD8^+^ T cell proliferation without the requirement for exogenous antigen stimulation. Taken together, these data demonstrate male antibodies generated to natural infection can enhance female infection during transmission, translocating infection across epithelial cells in the upper FRT and enhancing infection and activation of antigen‐presenting cells which rapidly migrate into draining lymph nodes, and enhance T cell differentiation.

The dynamics of *C. trachomatis* infection of antigen‐presenting cells; specifically, professional antigen‐presenting cells, including dendritic cells and macrophages, has been widely studied.^20^ The role of IgG‐opsonization on antigen presentation on chlamydial infection of antigen‐presenting cells is less well elucidated, but mouse macrophages with functional FcγR_I_ (CD64), FcγR_IIB1_ (CD32) and FcγR_III_ (CD16) have enhanced antibody‐dependent cellular cytotoxicity (ADCC) killing of infected cells and enhance Th1 responses in vitro, compared to FcγR‐deficient macrophages and antibody controls.^45^ In this study, we observed infection of both macrophages and dendritic cells. Opsonization of EBs with IgG enhanced viable (IncA^+^) chlamydial infection of dendritic cells, and enhanced in vitro proliferation of CD4^+^ CD8^+^ T cell proliferation of splenocytes from infected mice. Infection of APCs with opsonized EBs enhanced expression of chemokines CXCL1, 2, 5 and pro‐inflammatory cytokines IL1β and IFNγ and TNF mRNA. Similarly, antigen presentation and maturation of dendritic cell and macrophages infected with opsonized EBs significantly alters the transcriptome of the cells towards a pro‐inflammatory phenotype, which phenotypically enhances CD4^+^ and CD8^+^ T cell proliferation, that is, APC infected with opsonized *Chlamydia* rapid traffic to draining lymph nodes and express pro‐inflammatory markers, facilitating chlamydial‐specific T cell proliferation.

While opsonization of EBs with male IgG enhanced translocation and antigen presentation in vitro and in vivo, the kinetics of chlamydial shedding from the vagina of naïve mice remained unchanged, regardless of dose of IgG. However, and importantly in case of immunopathology, there was a significant and dose‐dependent enhancement of the incidence of oviduct occlusion/hydrosalpinx (infertility) in female mice infected with IgG‐opsonized *Chlamydia*. This phenomenon was chlamydial plasmid‐independent, and was associated with enhanced Th1 (IFNγ production) and doubling the proliferation of CD4^+^ and CD8^+^ T cells. This demonstrates that male IgG, at doses exceeding the upper limits of IgG concentration in ejaculate, is insufficient to prevent infection of naïve females, and can enhance immunopathology and infertility.

Surprisingly, the enhanced production of CD4^+^ and CD8^+^ T cells did not accelerate the vaginal clearance or total burden, suggesting that enhancing Th1 responses to whole *Chlamydia* does not necessarily support the clearance of the infection. This is in contrast to other studies finding that CD4^+^ T cells are necessary and sufficient to both clear infection, and prevent reinfection.^46^ This may suggest that the phenotype of Th1 cells generated plays a more important role than simply the numbers of CD4^+^ T cells generated or amount of IFNγ produced. While numerous studies (including our own) have identified an important role for CD4^+^ T cells in protection from pathology, particularly in the context of vaccination,^34^, ^47^ the enhancement of *Chlamydia*‐specific CD4^+^ T cells producing IFNγ in response to infection with opsonized EBs did not result in a reduced incidence of hydrosalpinx. However, oviduct occlusion may have occurred prior to the generation of sufficient numbers of protective *Chlamydia*‐specific CD4^+^ T cells.

Cytotoxic CD8^+^ T cells play a controversial role in chlamydial infections. While it would seem obvious that an obligate intracellular pathogen would be particularly vulnerable to CD8^+^ T cell‐mediated killing of infected cells (in the same way that many viral infections are), the chlamydial literature appears to suggest the opposite. Murthy and colleagues demonstrated that CD8^+^ T cells producing TNF in response to infection contribute significantly to hydrosalpinx formation,^48^ and they went on to demonstrate that minimal hydrosalpinx develops in OT‐I mice which have a CD8^+^ T cell repertoire limited to ovalbumin peptide (Ova_257‐264_).^49^ Hydrosalpinx in OT‐I mice could be reverted to that of WT mice when they received a pool of enriched naïve CD8^+^ T cells from WT mice.^49^ This strongly suggests *Chlamydia*‐specific CD8^+^ T cells generated in response to infection (and not non‐specific inflammation or cytotoxicity) contribute significantly to upper FRT pathology, leading this study to focus on CD8^+^ T cells for depletion.

CD8^+^ T cells typically respond to endogenous antigen presented by APCs via MHC‐I, but can also respond to exogenous (or phagocytosed) antigen presented by DCs through a process termed ‘cross‐presentation’. CD8‐CD11b^+^ DCs bind IgG:IC via FcγR, phagocytosing the ICs, which are acidified allowing intracellular FcRn to bind the IgG:IC and trafficking it to the proteasome for MHC‐I‐loading.^31^ This process facilitates enhanced CD8^+^ T cell proliferation and production of pro‐inflammatory effector functions including IFNγ, TNF and granzyme B production.^31^ In this study, the cross‐presentation and enhanced expansion of the immunopathogenic *Chlamydia*‐specific CD8 T cell pool could be greatly ameliorated in the absence of FcRn, and with prior depletion of the naïve CD8 T cell population.

The delivery of antigen/pathogens opsonized in IgG to APCs has long been known to enhance immunogenicity and killing of pathogens. Mucosal transport of IgG‐opsonized Ag across epithelial barriers provides the immune system the opportunity to rapidly respond to primary infection (e.g. when male IgG‐Ag is delivered to a naïve partner), or more commonly in recall immunity to secondary infection (self‐IgG rapidly delivering IgG‐bound Ag from the mucosal lumen and expanding memory T cells). This rapid augmentation of immunity allows delivery of Ag to APCs in the lamina propria without the need to disrupt the barrier integrity of the epithelium. FcγR‐mediated uptake of most pathogens leads to phagosolysosomal degradation and rapid killing. However, pathogens that are able to escape IgG/FcγR‐mediated phagocytosis and phagolysosomal degradation (e.g. *Chlamydia* spp.) can utilize this pathway as a Trojan horse to enhance infection and escape killing. Regardless, FcRn‐mediated transcytosis of luminal IgG‐bound pathogens is likely to be protective and thus beneficial in immunity in most circumstances consistent with the conservation of the *Fcgrt* gene across the class Mammalia. As shown here, *Chlamydia* spp. may be an exception to this rule.

This study supports mucosal delivery of antigen–antibody complexes has been demonstrated to enhance protective immune responses against infectious disease and tumours.^50^ Conjugation of the herpes simplex virus 2 (HSV2) glycoprotein D to the Fc fragment of IgG enhanced FcRn‐mediated transcytosis of HSV2 antigen to DCs in the lung, and together with the adjuvant CpG provided long‐term immunity from lethal challenge in mice. This may be an interesting approach for future studies in mucosal delivery of chlamydial antigens. Additionally, future experiments should utilize whole ejaculate from infected mice for a wholistic and physiologically relevant approach, including immune factors like complement which effect chlamydia viability.^51^, ^52^ This may help elucidate any potential clinical relevance and the mechanism for the 15%‐22% reduction in pathology of fertility observed in this study.

## Supporting information


Figure S1.

## Data Availability

The data that supports the findings of this study are available in the supplementary material of this article.
