# Supplementary material for: IgG exacerbates genital chlamydial pathology in females by enhancing pathogenic CD8 + T cell responses
Source: Scand J Immunol. 2023 Oct 13;99(1):e13331. doi: 10.1111/sji.13331 (PMC10909563; doi:10.1111/sji.13331)
Supplement: Supplementary file 1 — Figure S1. [file SJI-99-e13331-s001.docx]

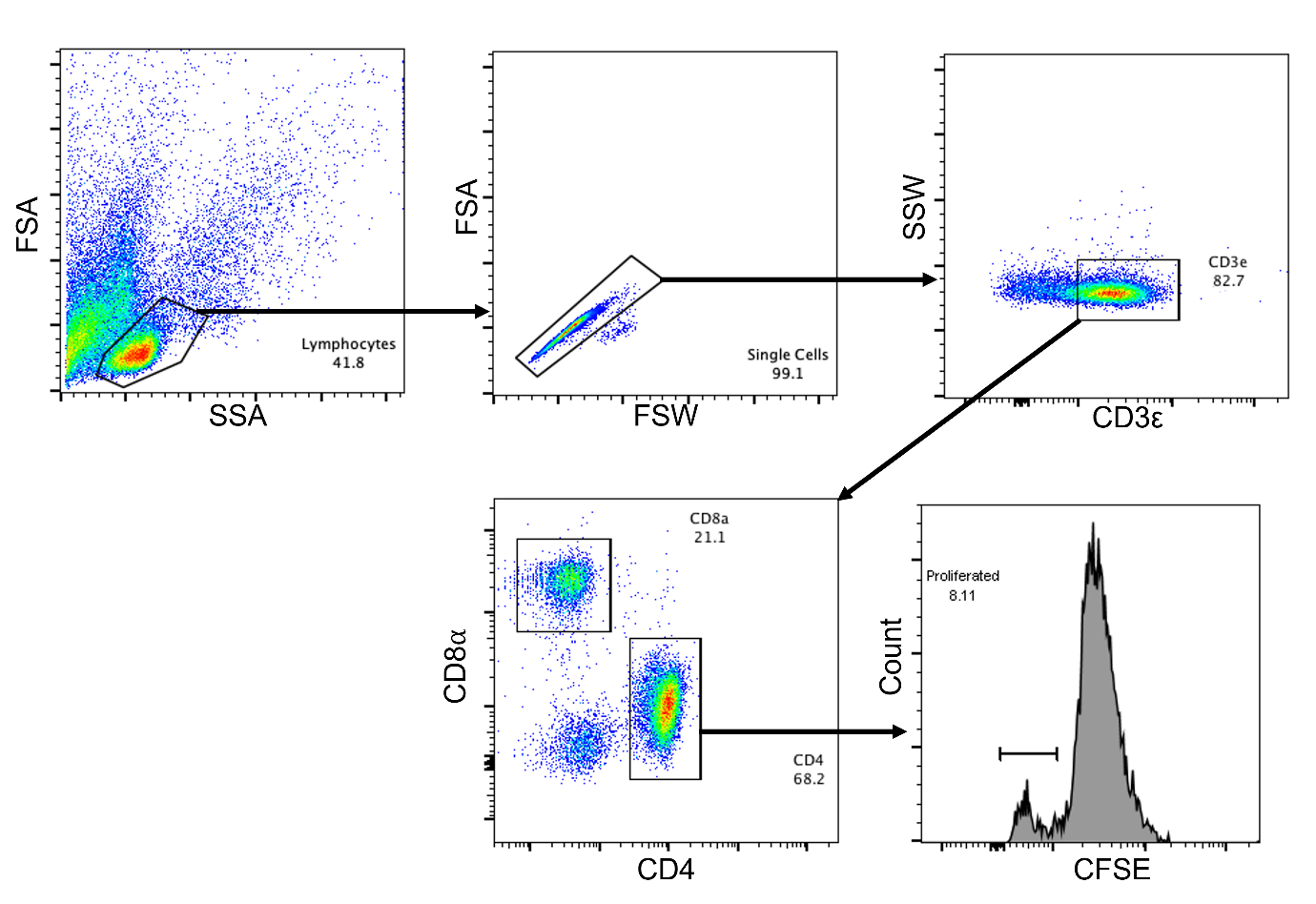


**Supplementary Figure 1:**

Gating strategy for CD4 T Cell proliferation. Representative flow gating strategy of cells collected from spleens and lymph nodes collected and stained with CFSE (to show proliferation) prior to stimulation with live *C. muridarum* or media controls for three days. Cells were then labelled with antibodies for CD3e, CD4, and CD8, fixed, and analysed by flow cytometry (BD FACS Aria III).
